# Protective Effect of Quercetin 3-*O*-Glucuronide against Cisplatin Cytotoxicity in Renal Tubular Cells

**DOI:** 10.3390/molecules27041319

**Published:** 2022-02-15

**Authors:** Daniel Muñoz-Reyes, Alfredo G. Casanova, Ana María González-Paramás, Ángel Martín, Celestino Santos-Buelga, Ana I. Morales, Francisco J. López-Hernández, Marta Prieto

**Affiliations:** 1 Toxicology Unit, Universidad de Salamanca, 37007 Salamanca, Spain; danimr@usal.es (D.M.-R.); alfredogcp@usal.es (A.G.C.); amorales@usal.es (A.I.M.); martapv@usal.es (M.P.); 2 Department of Physiology and Pharmacology, Universidad de Salamanca, 37007 Salamanca, Spain; 3 Group of Translational Research on Renal and Cardiovascular Diseases (TRECARD), 37007 Salamanca, Spain; 4 Institute of Biomedical Research of Salamanca (IBSAL), 37007 Salamanca, Spain; 5 National Network for Kidney Research REDINREN, RD016/0009/0025, Instituto de Salud Carlos III, 28029 Madrid, Spain; 6 Polyphenols Research Group (GIP-USAL), Nutrition and Bromatology Unit, Faculty of Pharmacy, Universidad de Salamanca, 37007 Salamanca, Spain; paramas@usal.es (A.M.G.-P.); csb@usal.es (C.S.-B.); 7 High Pressure Processes Group, BioEcoUVa, Bioeconomy Research Institute, Department of Chemical Engineering and Environmental Technology, Universidad de Valladolid, 47011 Valladolid, Spain; mamaan@iq.uva.es

**Keywords:** quercetin, quercetin 3-*O*-glucuronide, cisplatin, nephrotoxicity, cytoprotection

## Abstract

Quercetin, a flavonoid with promising therapeutic potential, has been shown to protect from cisplatin nephrotoxicity in rats following intraperitoneal injection, but its low bioavailability curtails its prospective clinical utility in oral therapy. We recently developed a micellar formulation (P-quercetin) with enhanced solubility and bioavailability, and identical nephroprotective properties. As a first aim, we herein evaluated the oral treatment with P-quercetin in rats, which displayed no nephroprotection. In order to unravel this discrepancy, quercetin and its main metabolites were measured by HPLC in the blood and urine after intraperitoneal and oral administrations. Whilst quercetin was absorbed similarly, the profile of its metabolites was different, which led us to hypothesize that nephroprotection might be exerted in vivo by a metabolic derivate. Consequently, we then aimed to evaluate the cytoprotective capacity of quercetin and its main metabolites (quercetin 3-*O*-glucoside, rutin, tamarixetin, isorhamnetin and quercetin 3-*O*-glucuronide) against cisplatin toxicity, in HK-2 and NRK-52E tubular cell lines. Cells were incubated for 6 h with quercetin, its metabolites or vehicle (pretreatment), and subsequently 18 h in cotreatment with 10–300 μM cisplatin. Immediately after treatment, cell cultures were subject to the MTT technique as an index of cytotoxicity and photographed under light microscopy for phenotypic assessment. Quercetin afforded no direct cytoprotection and quercetin-3-*O*-glucuronide was the only metabolite partially preventing the effect of cisplatin in cultured tubule cells. Our results identify a metabolic derivative of quercetin contributing to its nephroprotection and prompt to further explore exogenous quercetin-3-*O*-glucuronide in the prophylaxis of tubular nephrotoxicity.

## 1. Introduction

Cisplatin (cis-diaminnedichloroplatin (II)) is an inorganic compound with antineoplastic activity. Its pharmacological mechanism of action is based on the formation of a covalent bond with nitrogen 7 in purine bases, blocking cell division and inducing apoptosis [1]. As a chemotherapeutic drug, the use of cisplatin is curtailed by its toxicity, with nephrotoxicity posing the limiting effect [2]. The incidence of cisplatin nephrotoxicity is high, occurring in one in every three patients under treatment [3]. This nephrotoxicity is due to the drug accumulation in the kidney, since its elimination from the body is carried out through this organ by glomerular filtration and tubular secretion, without being metabolized. The copper transporter 1 (Ctr1) and the organic cation transporter 2 (OCT2) are mainly responsible for the entry of cisplatin into tubular renal cells, since they have a high affinity for the drug. In this way, its concentration in the epithelial cells of the proximal tubule is around 5 times higher than in plasma [4].

Several molecular mechanisms of cisplatin tubular cytotoxicity have been described, including: (a) direct DNA damage from adducts formations [5], (b) alteration of cellular transporters [6], (c) mitochondrial dysfunction and oxidative stress [5], (d) MAP kinase activation [7,8], (e) induction of apoptosis [3], and (f) inflammation [9]. These mechanisms constitute potential pharmacological targets for the prevention and alleviation of cisplatin nephrotoxicity [6].

Quercetin is a flavonoid found naturally in many fruits and vegetables. It has a wide variety of biological properties, among which its antioxidant, antiobesity, antiviral, antibacterial and anti-inflammatory effects stand out. Furthermore, quercetin is of special interest in the treatment of certain cancers, since it inhibits the proliferation of cancer cells and limits their growth [10].

In natural media, quercetin is usually found in glycosylated form. Quercetin glycosides can be partially hydrolyzed in saliva [11] and further in the small intestine by the enzyme lactase-phlorizin hydrolase in the intestinal epithelium releasing the aglycone that may be subsequently absorbed [12]. Efficient glucuronidation of quercetin can already occur in the small intestine by the action of UDP-glucuronyltransferases, as well as methylation by the action of catechol *O*-methyltransferases. Later, they are transported to the liver through the portal vein, where secondary metabolism occurs in the form of methylation, sulfation and conjugation with glucuronide. Conjugation of quercetin with sulfate is carried out by sulfotransferases. Thus, the circulating forms able to reach the biological targets are quercetin metabolites, which activity can differ from the original compound [13]. Once in the bloodstream, the metabolites bind to plasma proteins, such as albumin [10]. Finally, they accumulate in some organs, such as the kidney, which is involved in their excretion [14].

In the kidney, more than 90% of quercetin is in its conjugated form. Tubular cells have the enzymatic capacity to carry out a third biotransformation of quercetin. The metabolic conversions that occur include a complex combination of metabolite deconjugation followed by immediate sulfation, glucuronidation, methylation, and glucosylation [12].

We have previously demonstrated that quercetin protects against cisplatin nephrotoxicity in an in vivo model, without compromising the antineoplasic activity of the drug [3,15]. However, in our model, quercetin was administered intraperitoneally (i.p.), due to the low oral bioavailability of quercetin. The poor absorption and bioavailability of quercetin are mainly due to its limited solubility in aqueous fluids, which compromises the therapeutic application of the flavonoid. In order to improve its bioavailability, we have developed a micellar formulation (P-quercetin), which increases quercetin solubility approximately 10 times. Our results showed that this formulation, administered i.p., increased the plasma concentration of quercetin compared to the dose-equivalent administration of the unformulated flavonoid, and maintained the nephroprotective capacity when it was coadministered with cisplatin [16].

Since the i.p. via is not useful from a clinical point of view, and therefore the intended via of administration in humans is the oral one (p.o.), our first aim with this work was to verify whether P-quercetin protects against the nephrotoxicity of cisplatin by that via. However, the nephroprotection afforded by P-quercetin via i.p. was not observed following the administration of the same dosage through the oral via (see Section 2.1). In view of this result, a pilot study has been carried out to check whether the absorption of P-quercetin p.o. was lower than by the i.p., which might explain the absence of nephroprotection of the oral administration. With this objective, the presence of quercetin and its metabolites in biological samples (plasma and urine) was analyzed by HPLC, after the administration of the same dosage of P-quercetin by both routes. We found that P-quercetin was similarly absorbed via i.p. and p.o., whereas the profile of the metabolites found was slightly different between both vias (see Section 2.2). Our hypothesis in relation to these results is that quercetin in vivo protection may be related to the activity of some of its metabolite(s), and not to quercetin, at least partially. Thus, the pharmacokinetics after intraperitoneal administration would favor renal accumulation of the active metabolite/s to a greater extent than oral administration. So, the second aim of this study was to evaluate the cytoprotective capacity of quercetin and its main metabolites against cisplatin toxicity in renal tubular cells.

## 2. Results

### 2.1. Nephroprotection Study with Oral P-quercetin

**Figure 1:**
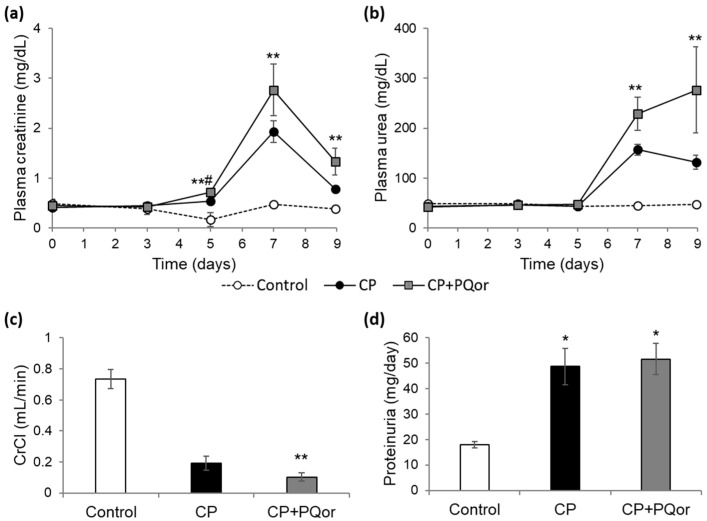
Evaluation of renal function in rats. (**a**) Evolution of plasma creatinine; (**b**) evolution of plasma urea; (**c**) creatinine clearance on day 7; (**d**) proteinuria on day 7. Values are expressed as the mean ± SEM. * *p* < 0.05; ** *p* < 0.01 vs. Control group; # *p* < 0.05 vs. CP group. CP: cisplatin (6.5 mg/kg, i.p.) on day 3; CP+PQor: P-quercetin (100 mg/kg, p.o.) for 9 days and cisplatin (6.5 mg/kg, i.p.) on day 3. CrCl: creatinine clearance.

In our in vivo model, renal function was heavily damaged by cisplatin. However, this effect was not ameliorated by oral P-quercetin, as was the case with intraperitoneal administration of the formulation in our previous experiments [16]. Rats in the cisplatin (CP) group experienced an acute kidney injury (AKI), as they showed a progressive increase in their plasma creatinine (Crpl) and urea levels compared to those of the controls (Figure 1a). AKI is defined and diagnosed according to elevations in Crpl concentration [17,18,19], an indirect marker of glomerular filtration rate (GFR). On the other hand, plasma urea concentration is a marker of azotemia [20]. These biomarkers also increased in the CP+PQor (cisplatin + oral P-quercetin) group, even slightly more extensive than in the CP group. In accordance with these data, cisplatin induced a severe drop in creatinine clearance (CrCl), a standard method for GFR measurement [21]. Oral P-quercetin also was unable of mitigate the drop in CrCl produced by cisplatin (Figure 1b). Finally, a significant increase in proteinuria was detected in both groups, CP and CP+PQor, on day 7. Although proteinuria may have glomerular origin, in the case of cisplatin nephrotoxicity it arise from defective tubular reabsorption due to tubular injury [22]. Oral P-quercetin also did not reverse in our model the excess urinary excretion of proteins produced by cisplatin (Figure 1b). The same pattern was found when twice the dose of P-quercetin was administered and the number of days of flavonoid administration prior to cisplatin was increased to 10 days (data not shown).

### 2.2. Metabolites Distribution in Plasma and Urine after P-quercetin Administration

**Figure 2:**
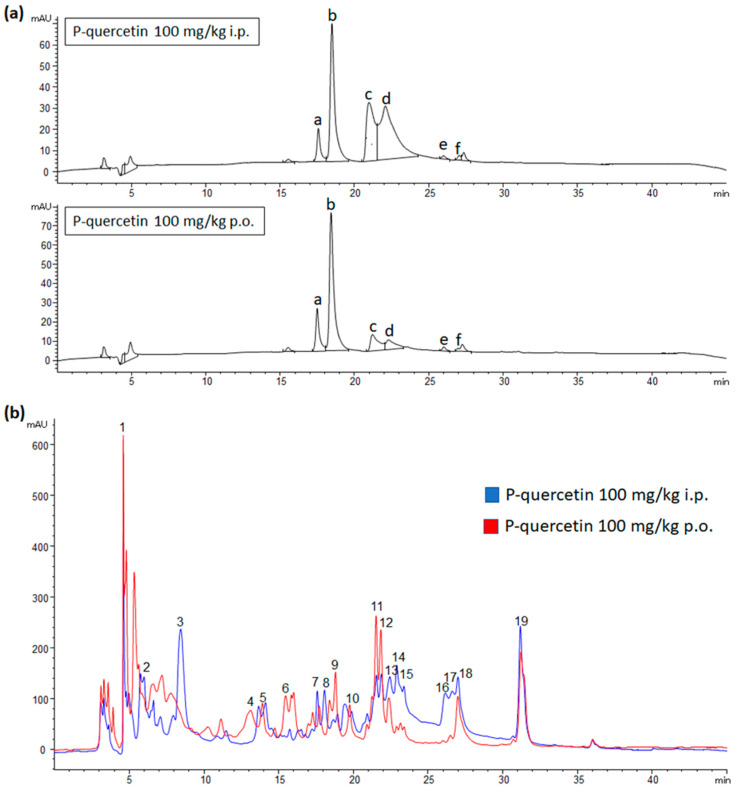
HPLC chromatograms recorded at 360 nm of plasma (**a**) and urine (**b**) samples from rats treated with P-quercetin. The formulation (100 mg/kg) was administered for 3 days, p.o. or i.p. Tentative identification of peaks: **a** and **b**: quercetin glucuronide sulfate; **c**: methylquercetin glucuronide sulfate; **d** and **e**: quercetin sulfate; **f**: methylquercetin sulfate. **1**: Protocatechuic acid derivative; **2**: hydroxyphenylacetic sulfate; **3** and **8**: quercetin glucuronide sulfate; **4**: protocatechuic acid; **5**: methylquercetin diglucuronide; **6**: quercetin sulfate derivative; **7**: quercetin diglucuronide + quercetin glucose; **9**: quercetin glucuronide; **10**: methylquercetin glucuronide sulfate; **11**: methylquercetin glucuronide + quercetin glucuronide sulfate; **12** and **15**: methylquercetin glucuronide; **13**, **14** and **16**: quercetin sulfate; **17** and **18**: methylquercetin sulfate; **19**: methylquercetin. Information on MS data of the peaks is given in Table S1, Supplementary Material.

The distribution of quercetin and its metabolites was analyzed in the plasma and urine of rats that had been administered 3 doses of P-quercetin, i.p. or p.o. In the case of plasma samples, a fairly similar profile in the chromatogram was obtained after flavonoid administration for each of the vias. This fact indicates that absorption occurred by both vias. However, differences were observed in the levels of some metabolites, namely methylquercetin glucuronide sulfate and quercetin sulfate, which appeared in higher concentrations after i.p. than after oral administration (Figure 2a). The metabolites profile found in urine samples was more complex than the one observed in plasma, with a greater number of quercetin metabolites detected. In this case, differences, mainly quantitative, were observed in some metabolites depending on the via of administration (Figure 2b), but also on the individuals.

### 2.3. Evaluation of the Protective Capacity of Quercetin and Its Metabolites against Cisplatin Cytotoxicity in HK-2 Cells

#### 2.3.1. Titration of Cisplatin in HK-2 Cells

Cisplatin cytotoxicity was determined in HK-2 cells, a cell line of human proximal tubule cells [23].

**Figure 3:**
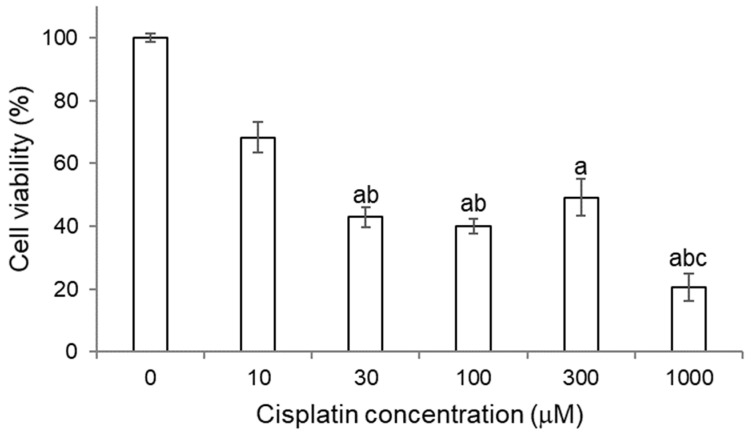
Titration of cisplatin cytotoxicity for 18 h of treatment in HK-2 cells. Cell viability was determined using the MTT assay. Values are expressed as the mean ± SEM. Significant differences (*p* < 0.05): a vs. 0 μM; b vs. 10 μM; c vs. 300 μM.

As it can be observed in Figure 3, a decrease in the cell viability of HK-2 cells was produced as the concentration of cisplatin increased. At 10 μM, a decrease in cell viability of approximately 30% was already observed compared to cells without treatment, but the difference was not statistically significant. From a concentration of 30 μM, a statistically significant decrease in cell viability was observed, indicating the presence of cell damage at such concentrations of cisplatin for HK-2 cells.

Microscopic images of HK-2 cells treated with different doses of cisplatin confirmed the presence of cell damage, as manifested in cell viability assays (MTT). Compared with the Control group, at a concentration of 10 μM of cisplatin, no cell damage was observed, so this concentration seems to exert an antiproliferative effect (according to MTT). At a concentration of 30 μM of cisplatin, apoptotic bodies and cisplatin-induced changes in cell morphology began to be detected. On the other hand, at 300 μM, necrosis was observed. The cellular phenotype after cisplatin treatment can be a key when evaluating the possible cytoprotection of quercetin metabolites. These results confirm those previously obtained by Sancho-Martínez et al. [5].

#### 2.3.2. Titration of Quercetin and Its Metabolites in HK-2 Cells

In order to know the highest non-toxic concentrations for quercetin and its metabolites, the corresponding cell safety experiments were performed. Concentrations between 0.4–200 μM (Figure S1, Supplementary Material) were tested. The results are summarized in Table 1.

Our results indicated that the highest metabolite concentration not toxic to cells is different among compounds of the same group, such as rutin and quercetin 3-*O*-glucoside, which are glycosylated compounds. Furthermore, quercetin 3-*O*-glucoside was the most toxic metabolite of those tested. On the other hand, rutin and quercetin 3-*O*-glucuronide were the compounds with a greater safety margin, being the concentration of 25 μM, in both cases, the highest non-toxic.

#### 2.3.3. Protection Assays of Quercetin and Its Metabolites against Cisplatin Cytotoxicity in HK-2 Cells

Next, experiments were carried out to test the efficacy of quercetin and its metabolites against the damage of cisplatin. Cytotoxic concentrations of the drug were used according to the viability experiments and the images obtained in the light microscope (see Section 2.3.1): antiproliferative (10 μM), apoptotic (30 μM) and necrotic (300 μM) effect. On the other hand, the highest non-toxic concentration of each metabolite was used for cotreatment with cisplatin, according to Table 1.

**Figure 4:**
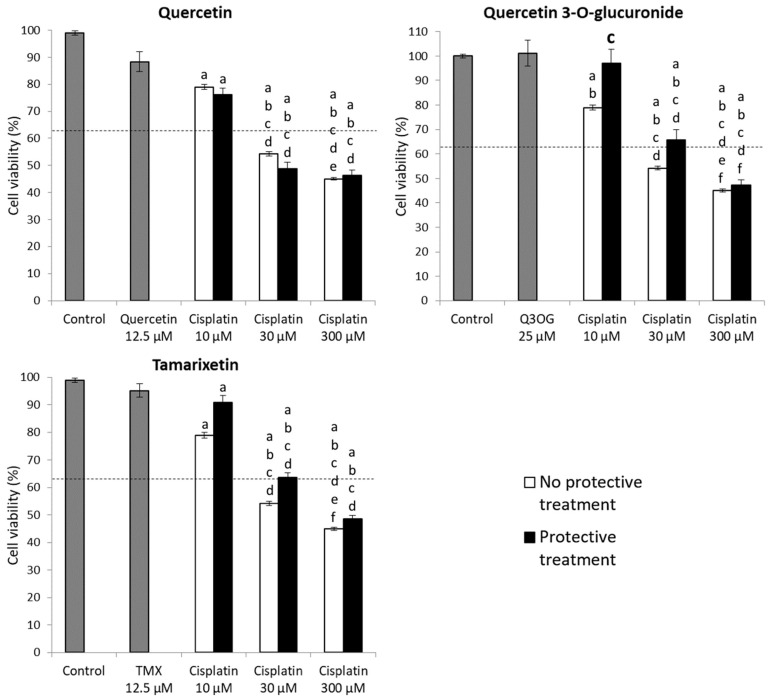
Quercetin, quercetin 3-*O*-glucuronide and tamarixetin efficacy assays in HK-2 cells for 24 h against cisplatin cytotoxicity. The cells were pretreated 6 h with the corresponding metabolite and subsequently cotreated for 18 h with the metabolite and cisplatin 10, 30 and 300 µM, respectively. The MTT technique was used to estimate cell viability. Values are expressed as the mean ± SEM. Significant differences (*p* < 0.05): a vs. Control; b vs. Metabolite alone (quercetin, quercetin 3-*O*-glucuronide or tamarixetin); c vs. 10 µM cisplatin; d vs. cisplatin 10 µM + protective treatment; e vs. 30 µM cisplatin; f vs. cisplatin 30 μM + protective treatment. The dotted line represents cell viability before treatment (time 0 h). Q3OG: quercetin-3-*O*-glucuronide; TMX: tamarixetin.

HK-2 cells were subjected to 6-h pretreatments with quercetin/quercetin metabolites and then 18-h cotreatment with cisplatin and the corresponding metabolite. The results obtained for quercetin, quercetin 3-*O*-glucuronide and tamarixetin are shown in Figure 4. The results for the rest of the metabolites (quercetin 3-*O*-glucoside, isorhamnetin and rutin) can be consulted in Figure S2 (Supplementary Material).

Quercetin, at the dose tested (12.5 μM), did not affect the viability of HK-2 cells. However, its cotreatment with cisplatin was not effective in reversing the decrease in the number of viable cells caused by the drug. Therefore, according to our results, quercetin does not prevent the cytotoxicity of cisplatin in HK-2 cells (Figure 4).

Regarding the action of the glycosylated metabolites, quercetin 3-*O*-glucoside (0.8 μM) and rutin (25 μM), in cotreatment with cisplatin, no differences were observed with respect to the treatments only with cisplatin at any of the doses tested for the cytotoxic agent (Figure S2).

**Figure 5:**
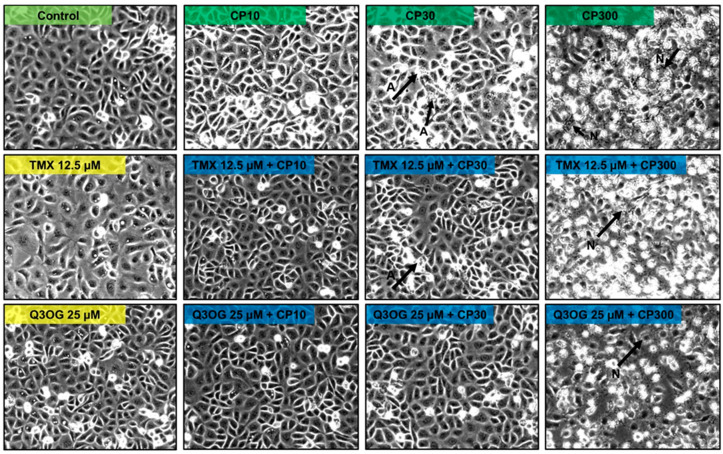
Representative images of HK-2 cells taken under the light microscope (10× magnification). First line: cells without treatment (Control) and treated with cisplatin 10 (CP10), 30 (CP30) and 300 (CP300) μM for 18 h. Second line: cells treated with tamarixetin (TMX) at a concentration of 12.5 μM for 24 h, and cells pretreated with TMX 12.5 μM for 6 h and subsequently cotreated 18 h with TMX 12.5 μM and CP10, CP30 and CP300, respectively. Third line: cells treated with quercetin 3-*O*-glucuronide (Q3OG) at a concentration of 25 μM for 24 h, and cells pre-treated with Q3OG 25 μM for 6 h and subsequently cotreated for 18 h with Q3OG 25 μM and CP10, CP30 and CP300, respectively. TMX: tamarixetin; G3OG: quercetin 3-*O*-glucuronide; CP: cisplatin; A: apoptosis; N: necrosis.

Regarding the methylated metabolites, the cotreatment of the cells with tamarixetin (12.5 μM) originated an increment on the cell viability profile in cells treated with cisplatin at the three doses tested. Moreover, photos of the cell culture were taken under light microscope, showing that cells cotreated with tamarixetin and cisplatin at doses 10 and 30 μM presented a better status than those treated with the same dose of cisplatin itself (Figure 5). However, these differences were not significant with respect to the single antineoplastic treatment at each of the concentrations (Figure 4). Instead, cotreatment with isorhamnetin (1.56 μM) did not protect from the decrease in cell viability produced by cisplatin; by contrast, an enhancing effect of isorhamnetin on the cytotoxicity of cisplatin was even observed, although the data were not statistically significant (Figure S2).

When the cells were pretreated with quercetin-3-*O*-glucuronide and, subsequently, cotreated with the metabolite and cisplatin at doses of 10 and 30 μM, an increase in cell viability of, respectively, 20 and 10% was observed, compared to treatments with cisplatin without metabolite (Figure 4). This result indicates that cotreatment with quercetin 3-*O*-glucuronide induces cellular protection, thus avoiding or reducing the damage caused by cisplatin. In fact, photos of the cell culture showed that cells cotreated with quercetin 3-*O*-glucuronide and cisplatin, at the three doses tested, presented a better status than those treated with the same dose of cisplatin itself (Figure 5). However, in the second case (cotreatment with cisplatin 30 μM) the results were not statistically significant, although a cytotoprotection profile was observed.

### 2.4. Evaluation of the Protective Capacity of Quercetin and Its Metabolites against Cisplatin Cytotoxicity in NRK-52E Cells

Considering the results obtained in HK-2 cells for quercetin 3-*O*-glucuronide (see Section 2.3), it was decided to corroborate the cytoprotective effect of the metabolite in a different tubular cell line. Thus, the NRK-52E cell line was used for several reasons: first of all, they are cells from rats, which allowed us to evaluate the effect of quercetin 3-*O*-glucuronide in a different species (HK-2 cells come from human kidney). Second, they are renal epithelial cells [24], so they have a less specific origin than HK-2 cells, which are defined as proximal tubule cells [23].

The experiments with NRK-52E cells were carried out with only three compounds: quercetin (reference molecule in our experiments), quercetin 3-*O*-glucuronide and tamarixetin. The latter was introduced based on the good cytoprotective tendency observed in the experiments with HK-2 cells (see Section 2.3.3).

#### 2.4.1. Titration of Cisplatin in NRK-52E Cells

In the same way, the cytotoxicity of cisplatin was determined in NRK-52E cells. The aim was to find the cisplatin concentrations that gave rise to the same phenotypes found in HK-2 cells (antiproliferative, apoptotic and necrotic effects) via MTT assay and light microscope photos, to subsequently perform cytoprotection experiments with these concentrations.

**Figure 6:**
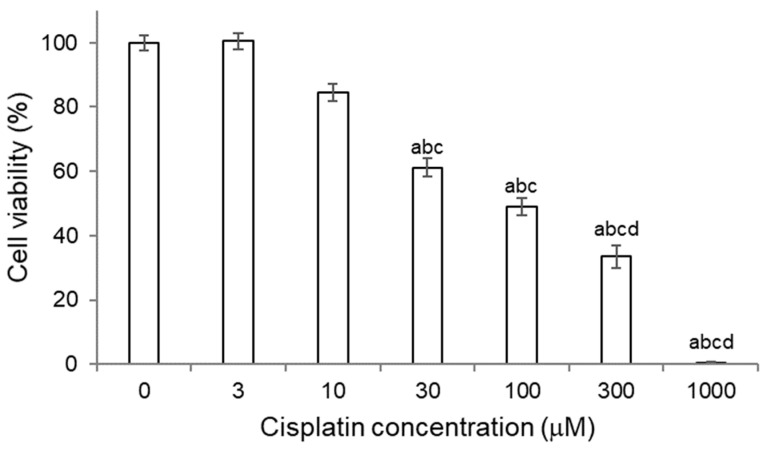
Titration of cisplatin cytotoxicity for 18 h of treatment in NRK-52E cells. Cell viability was determined using the MTT assay. Values are expressed as the mean ± SEM. Significant differences (*p* < 0.05): a vs. 0 μM, b vs. 3 μM; c vs. 10 μM; d vs. 30 μM.

A decrease in the viability of NRK-52E cells was observed (Figure 6) as the cisplatin concentration increased, quite similar to what we had previously observed for HK-2 cells (see Section 2.3.1). At the 10 μM concentration of cisplatin, a decrease in cell viability of approximately 20% compared to cells without treatment was found, but the difference was not statistically significant compared to the control. From 30 µM, a statistically significant decrease in cell viability was observed, indicating the presence of cell damage at such concentrations of cisplatin for NRK-52E cells.

The images of NRK-52E cells treated with different doses of cisplatin confirmed the presence of cellular damage, observing a similar phenotype to that of HK-2 cells at the same doses: cisplatin 10 μM concentration did not produce apparent changes in cell morphology, for which it seems to exert an antiproliferative effect according to MTT, while at a concentration of 30 μM of cisplatin, the formation of apoptotic bodies began to be observed. At 300 μM, death by necrosis was observed.

#### 2.4.2. Titration of Quercetin and Its Metabolites in NRK-52E

In order to know the highest non-toxic concentrations for each metabolite in NRK-52E cells, cell safety experiments were assessed. Doses between 0.4–200 μM (Figure S3, Supplementary Material) were tested for each metabolite. The results are summarized in Table 2.

The data indicated that tamarixetin was the most toxic metabolite of those tested, while quercetin had an intermediate cellular safety range. On the other hand, quercetin 3-*O*-glucuronide was the compound that revealed the highest safety range, being 200 μM the highest non-toxic concentration of those evaluated. It is noteworthy that the safety range of the glucuronide was 4 times higher than in HK-2 cells.

#### 2.4.3. Protection Assays of Quercetin and Its Metabolites against Cisplatin Cytotoxicity in NRK-52E Cells

Efficacy experiments were carried out in NRK-52E cells to check whether quercetin metabolites had a protective effect against cisplatin damage. Cytotoxic cisplatin concentrations were used according to the viability experiments (MTT) and the images obtained under the light microscope: antiproliferative (10 μM), apoptotic (30 μM) and necrotic (300 μM) effect. On the other hand, the highest non-toxic concentration of each metabolite was used for cotreatment with cisplatin, according to Table 2.

**Figure 7:**
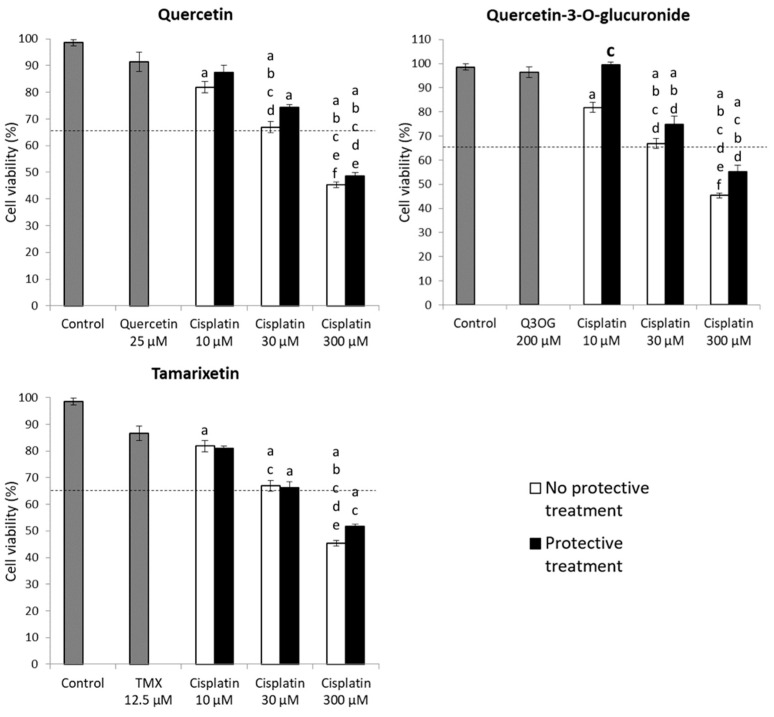
Quercetin, quercetin 3-*O*-glucuronide and tamarixetin efficacy assays in NRK-52E cells for 24 h against the cytotoxicity of cisplatin. The cells were pretreated for 6 h with the corresponding metabolite and subsequently co-treated for 18 h with the metabolite and 10, 30 and 300 μM cisplatin, respectively. The MTT technique was used to estimate cell viability. Values are expressed as the mean ± SEM. Significant differences (*p* < 0.05): a vs. Control; b vs. Protective treatment alone; c vs. 10 µM cisplatin; d vs. Cisplatin 10 µM + Protective treatment; e vs. 30 µM cisplatin; f vs. Cisplatin 30 μM + Protective treatment. The dotted line represents the state of the cells before treatment (time 0 h). Q3OG: quercetin-3-*O*-glucuronide; TMX: tamarixetin.

**Figure 8:**
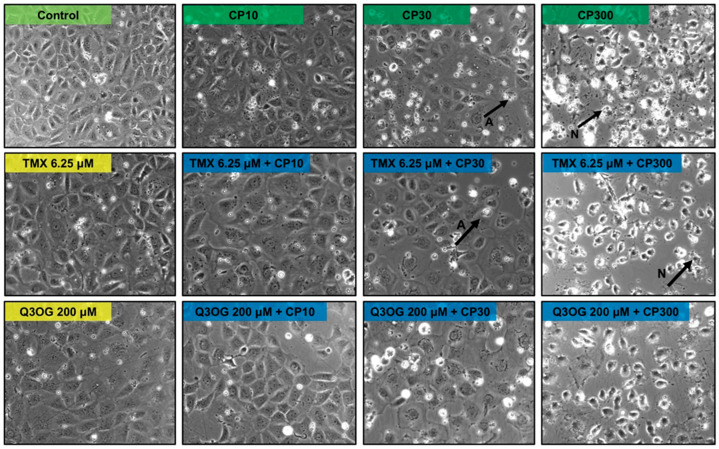
Representative images of NRK-52E cells taken under the light microscope (10× magnification). First line: cells without treatment (control) and treated with cisplatin 10 (CP10), 30 (CP30) and 300 (CP300) μM for 18 h. Second line: cells treated with tamarixetin (TMX) at a concentration of 12.5 μM for 24 h, and cells pre-treated with TMX 12.5 μM for 6 h and subsequently cotreated 18 h with TMX 12.5 μM and CP10, CP30 and CP300, respectively. Third line: cells treated with Quercetin 3-*O*-glucuronide (Q3OG) at a concentration of 25 μM for 24 h, and cells pre-treated with Q3OG 25 μM for 6 h and subsequently cotreated for 18 h with Q3OG 25 μM and CP10, CP30 and CP300, respectively. TMX: tamarixetin; Q3OG: quercetin 3-*O*-glucuronide; CP: cisplatin; A: apoptosis; N: necrosis.

A 6-h pretreatment with quercetin/quercetin metabolite was performed, followed by an 18-h cotreatment with cisplatin and the corresponding compound. The results are presented in Figure 7 and Figure 8.

Cotreatment with quercetin 25 μM and cisplatin resulted in a mild increase in cell viability for the three concentrations of cisplatin tested, compared to the single treatment with the drug, although the differences were not significant. On the other hand, cotreatment with tamarixetin and cisplatin made no difference compared to treatment without the metabolite. Therefore, tamarixetin does not appear to protect against drug-induced damage in our experiments with NRK-52E cells (Figure 7).

As with HK-2 cells, in NRK-52E cells the most interesting results were found with quercetin 3-*O*-glucuronide. When cells are pretreated and subsequently cotreated with the metabolite in combination with cisplatin at concentrations of 10, 30 and 300 μM, an increase in cell viability of 20, 10 and 10%, respectively, was observed, in comparison with treatments without metabolite. This result indicates that glucuronide cotreatment at a concentration of 200 μM induces cellular protection, thus avoiding or reducing the damage caused by cisplatin. Nevertheless, the results were not statistically significant for the cotreatment with cisplatin 30 and 300 μM and glucuronide. Therefore, it can be stated that quercetin 3-*O*-glucuronide, according to our results, protects against cisplatin damage partially (Figure 7 and Figure 8).

## 3. Discussion

In our experiments, quercetin 3-*O*-glucuronide induced cellular protection in the two tubular cell lines assessed, HK-2 and NRK-52E, since it was able to palliate the cytotoxic effect of cisplatin. In fact, the drug produced an antiproliferative effect at 10 μM concentration, which was reversed by the cotreatment with the quercetin metabolite. In contrast, at higher cisplatin concentrations (30 and 300 μM), no statistically significant results were obtained, although a cytoprotective trend was also observed at these concentrations. Therefore, the glucuronide appears to exert a moderate cytoprotective effect, preventing the cytotoxic effect caused by cisplatin at antiproliferative drug concentrations, and reducing the cell death caused by the drug at higher concentrations partially.

Based on those results, quercetin 3-*O*-glucuronide could be responsible, at least in part, for the nephroprotective effect of quercetin against cisplatin damage observed in vivo [3,15]. Furthermore, the protective effect of the metabolite could explain our in vivo results regarding to the absence of protection of oral P-quercetin, when the same formulation has previously been shown to be effective intraperitoneally [16]. The differences observed between the administration of P-quercetin by the intraperitoneal and oral vias cannot be explained by its absorption, since it seems to be very similar for both routes. However, plasma concentration of some quercetin metabolites was different, which might be explained by possible pharmacokinetic differences between both vias [25]. After i.p. administration, metabolism in enterocytes does not take place [12], while a first-pass effect occurs via p.o., with the compound being biotransformed before entering systemic circulation [26]. Therefore, the nephroprotection of quercetin against cisplatin could be related to the concentrations reached in the kidney by quercetin itself or its metabolites, which could be different depending on the administration route of the flavonoid. A relevant metabolite could be quercetin 3-*O*-glucuronide, according to our in vitro results.

Quercetin 3-*O*-glucuronide is one of the most common quercetin metabolites produced by phase II biotransformation in small intestine and liver cells, besides sulfated and methylated derivatives [27]. Once in the kidney, the entry of quercetin metabolites in tubular cells takes place mainly through influx transporters in the basolateral membrane and transporters in the apical membrane via tubular reabsorption [28]. It has been reported that glucuronide conjugates, such as quercetin 3’-*O*-glucuronide, have a high affinity for OAT3, whereas quercetin 3-*O*-glucuronide and quercetin 7-*O*-glucuronide are weak substrates of OAT1 and OAT3 [29]. Thus, it can be speculated that the use of quercetin 3’-*O*-glucuronide might still improve the cytoprotective effect observed for quercetin 3-*O*-glucuronide in the present work. It has also been suggested that these metabolites could be involved in the induction of antioxidant defense mechanisms through inducing the expression of antioxidant enzymes [12]. In addition, some published studies have demonstrated therapeutic effects of glucuronide metabolites [30,31]. In the kidney, an organ where metabolites accumulate to be eliminated, some glucuronides have been shown to have a biological effect. For example, they can reduce oxidative stress [32] or hypoxia signals in kidney cells [33]. On the other hand, other glucuronides behave as active compounds, increasing the prodrug toxicity [34,35]. Therefore, the biological activity of some glucuronide metabolites has been previously demonstrated. All in all, quercetin 3-*O*-glucuronide would reach kidney tissue through systemic distribution [36], and could exert a cytoprotective effect at the tubular level as indicated by our results. Nonetheless, the mechanisms by which the glucuronide exerts its protective effects from cisplatin toxicity must still be unveiled.

Cotreatment of quercetin aglycone, glycosylated derivatives (quercetin 3-*O*-glucoside and rutin), or methylated derivatives (tamarixetin and isorhamnetin) with cisplatin did not protect against drug cytotoxicity in our experiments. However, quercetin glycosides are present in human urine samples after ingesting foods rich in quercetin, whereas they are not present in plasma. This implies that they are formed in the kidney in situ based on local metabolism [12]. It should not be ruled out that glycosides are involved in nephroprotection, perhaps by action on other renal cells.

It either cannot be ruled out that aglycone could be responsible for the cytoprotective effect, since there are deconjugation processes in the tubular cell to give rise to aglycone [10]. Indeed, it has been reported that conjugation is a reversible process and glucuronides, but not sulfates, can be deconjugated at tissue level, yielding the parent aglycone, which could be the actual active form [37,38]. In fact, the tubular cells present all the enzymatic machinery to carry out a third biotransformation of quercetin in the kidney, which may contribute to the final concentrations reached in the kidney of quercetin and its metabolites. The high β-glucuronidase activity in tubular epithelial cells can, thus, be responsible for the deconjugation of glucuronides, to give rise to quercetin aglycone [12]. However, it has also been described that this deglucuronidation could be followed by instantaneous sulfation, and subsequent reglucuronidation [10]. This is consistent with the idea that glucuronide derivatives (and specifically quercetin 3-*O*-glucuronide) can be responsible, at least in part, for the protective effects of quercetin in the tubular cell.

The obtained results are very similar in both tubular cell lines, HK-2 and NRK-52E, despite the fact that, theoretically, there are some differences between them. HK-2 are defined as proximal tubule cells, while NRK-52E are renal epithelial cells, so they have a less specific origin. These differences could explain the results obtained with tamarixetin, a metabolite that seems to exert a certain cytoprotective action in the proximal tubule (HK-2), while this effect could be masked when carrying out the experiments with NRK-52E. However, this hypothesis needs to be analyzed more deeply.

Taking into account that the cytoprotective effect observed for quercetin 3-*O*-glucuronide in our experiments is partial and that quercetin metabolism is very complex [12], it is conceivable that the nephroprotective effect of quercetin observed in vivo could be the result of the joint action of several metabolites and on other targets in addition to the tubular cell. In fact, besides tubular protection, beneficial effects of quercetin have also been demonstrated at the hemodynamic level [39,40], which contribute to the blood supply of the kidney and the maintenance of the glomerular filtration rate when the flavonoid is coadministered with cisplatin [3]. In addition, according to in vitro studies, vasodilator effect can be exerted by both quercetin and isorhamnetin [41,42], which supports the idea that the metabolites could contribute to the in vivo effects of quercetin.

A strategy for future nephroprotection experiments could consist of administrating quercetin 3-*O*-glucuronide p.o. The administration of the glucuronide metabolite would facilitate access to the tubular cell, as well as its renal metabolism and accumulation. Although we are aware that quercetin glucuronide has shown a moderate cytoprotective effect, this strategy could be a further advance in the nephroprotective application of quercetin.

## 4. Materials and Methods

### 4.1. Animals and Bioethics

All procedures were approved by the Bioethics Committee of the University of Salamanca and the Regional Government of Castile and Leon, Ministry of Agriculture and Livestock (code: 0000075, 29 April 2016). Animals were handled according to the guidelines of the European Community Council Directive 2010/63/UE and to the current Spanish legislation for experimental animal use and care (RD 53/2013, 1 February 2013). Male Wistar rats (200–250 g) were maintained under controlled environmental conditions, with free access to water and standard chow.

### 4.2. Nephroprotection Study with Oral P-quercetin

**Figure 9:**
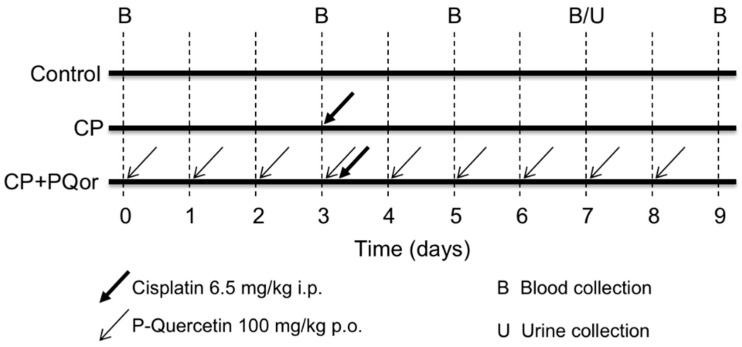
Scheme of the nephrotoxicity model. CP: cisplatin (6.5 mg/kg, i.p.) on day 3; CP + PQor: P-quercetin (100 mg/kg, p.o.) for 9 days and cisplatin (6.5 mg/kg, i.p.) on day 3.

A cisplatin nephrotoxicity model previously developed in our laboratory was used [16]. Rats were divided into three experimental groups (Figure 9): Control (*n* = 3), animals received vehicle (water) orally through a intragastric gavage (p.o.) for 9 days; CP (*n* = 5), animals received water p.o. for 9 days and a single nephrotoxic dose of cisplatin in NaCl 0.9% (6.5 mg/kg, i.p.) (Merck, Darmstadt, Germany) on day 3 of the experiment; and CP+PQor (*n* = 6) animals received a daily dose of P-quercetin (100 mg/kg, p.o., through a intragastric gavage (i.e., containing 50 mg/kg quercetin)) for 9 days and a dose of cisplatin in NaCl 0.9% (6.5 mg/kg, i.p.) on day 3. P-quercetin is a micellar formulation of quercetin, prepared as described previously [43].

Blood samples (150 μL) were collected on days 0, 3, 5, 7 and 9 in heparinized capillaries from a small incision in the tail tip. Plasma was separated by centrifugation (11,000 rpm for 3 min) and kept at −80 °C. On day 7 (time of maximum nephrotoxicity), 24 h urine was collected in metabolic cages, cleared by centrifugation (2000× *g* for 9 min) and stored at −80 °C. At the end of the experiment (day 9), rats were anesthetized and sacrificed by exsanguination.

### 4.3. Analysis of Quercetin and Its Metabolites in Blood and Urine Samples

A pilot assay was carried out in order to analyze the presence of quercetin and its metabolites in biological samples after the administration of P-quercetin orally and i.p. Two rats were treated with P-quercetin, p.o, at the dose of 100 mg/kg/day, for 3 days. Two other rats were simultaneously treated with the same dose, but administering the formulation i.p. On the fourth day, blood and urine (24 h) samples were collected and the rats were sacrificed by exsanguination. Blood and urine samples were processed as described in Section 4.2.

For HPLC analysis, 100 μL of plasma was taken and 300 μL of methanol was added. It was vortexed for one minute and centrifuged at 12,000 rpm for 10 min. Subsequently, the supernatant was collected, brought to dryness in the speedvac and the residue was redissolved in 120 μL of acetonitrile: 0.1% formic acid (20:80). In the case of urine samples, the protocol proposed by Mullen et al. [44] was followed, based on direct urine analysis. 200 μL of urine was taken and 10 μL of methanol was added in order to precipitate proteins. The samples were then shaken, centrifuged and injected into the HPLC system. Analysis were carried out by HPLC-DAD-ESI-MS as described elsewhere [45]. A Hewlett-Packard 1200 chromatograph (Agilent Technologies, Waldbronn, Germany) connected to an API 3200 138 Qtrap (Applied 139 Biosystems, Darmstadt, Germany) mass spectrometer was used. For detection, 280, 330 and 370 nm were selected as preferred wavelengths for the DAD and the MS was operated in the negative ion mode, spectra were recorded between *m/z* 100 and *m/z* 1500. The phenolic metabolites were identified by using data reported from literature and by comparison with our database library. All samples were analyzed in duplicate.

### 4.4. Renal Cells

Kidney tubular cells were used: HK-2 cells (human kidney 2), it is an immortalized proximal tubule cell line obtained from healthy human kidney (Ref: ATCC, CRL-2190), and NRK-52E (normal rat kidney), is an immortalized cell line of epithelial origin obtained from the rat (Ref: ATCC, CRL-1571). The culture medium for the HK-2 used was RPMI-1640 (Merck, Darmstadt, Germany), supplemented with fetal bovine serum (FBS) (10%) (ThermoFisher, Waltham, MA, USA), L-glutamine (1 mM) (Merck, Darmstadt, Germany) and penicillin-streptomycin (500 U/mL) (Merck, Darmstadt, Germany). For the NRK-52E, a DMEM medium was used: F12 with HEPES, glucose and L-glutamine (Lonza, Basel, Switzerland), supplemented with FBS (10%) and penicillin-streptomycin (500 U/mL).

All cell culture procedures were performed in a laminar flow hood using sterile material. The cells were incubated at 37 °C in a humid atmosphere, with 5% CO_2_.

### 4.5. Quercetin Metabolites

**Figure 10:**
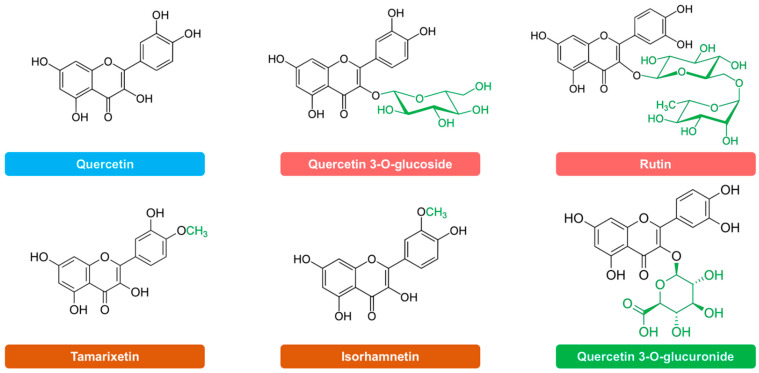
Chemical structure of quercetin and its metabolites used in this study.

Quercetin and its metabolites isorhamnetin, tamarixetin, quercetin 3-*O*-glucoside, rutin and quercetin 3-*O*-glucuronide (Figure 10), were used in the experiments carried out. All compounds were from Merck (Darmstadt, Germany) with the exception of quercetin (Acros Organics, Madrid, Spain) and tamarixetin (Cymit Química, Barcelona, Spain).

All of them were dissolved in dimethylsulfoxide (DMSO) and stored at −80 °C, in order to have a 0.1 M stock solution.

### 4.6. Design of Experiments In Vitro

24-well plates (15.6 mm diameter) were used to carry out the experiments. After trypsinize and counting the cells, 35,000 cells were seeded per well. Once they had adhered and were at 70% confluence, the experiments were started. The final volume in each well was 500 µL. In each experiment, four duplicates (wells) were made for each experimental condition.

#### 4.6.1. Titration of Cisplatin

Cisplatin (Merck, Darmstadt, Germany) titration in both cell types was performed as previously described [5]. Three experiments were performed for each tested cisplatin concentration (0–1000 µM). For the preparation of the solutions at different concentrations, a 1 M stock solution was started, and subsequently serial dilutions were made. After 18 h of treatment, the MTT test was carried out to determine cell viability (see Section 4.6).

#### 4.6.2. Titration of Quercetin and Its Metabolites

In order to establish the highest non-toxic concentration of each metabolite, four titration experiments were carried out for each cell type and metabolite, adjusting the concentrations according to the results that were obtained. For the preparation of solutions at different concentrations of each metabolite, a 0.1 M stock solution (in DMSO) was started, and serial dilutions were made. The highest concentration tested for each metabolite was 200 µM. After 24 h of treatment, the 3-(4,5-dimethylthiazol-2-yl)-2,5-diphenyltetrazolium bromide (MTT) test was carried out to determine cell viability (see Section 4.6).

In the case of NRK-52E cells, the metabolites to be tested were selected based on the results obtained in HK-2 cells.

In each experiment, a plate of cells was seeded to which the MTT assay was performed at the time of starting the treatments (time 0).

#### 4.6.3. Efficacy Experiments of Quercetin and Its Metabolites against Cisplatin Cytotoxicity

To check whether quercetin and/or its metabolites protected against the cytotoxicity of cisplatin, three concentrations of the drug were used: 10, 30 and 300 μM. These concentrations were chosen based on the results obtained in the titration experiments. Regarding quercetin and its metabolites, the highest non-toxic doses were selected for each cell type. Four experiments were performed for each cell type and metabolite.

A 6-h pretreatment was carried out with the quercetin metabolites and then, without removing the medium, cisplatin was added for 18 h. Once the incubations had elapsed, images were obtained by light microscope of each experimental condition and, subsequently, the MTT protocol was followed (see Section 4.6).

### 4.7. MTT Assay

As an index of cell viability, the MTT test was carried out. To each well was added, without removing the medium, 50 μL of MTT, (0.5 mg/mL) (Merck, Darmstadt, Germany) for 4 h at 37 °C (MTT concentration in the well 0.05 mg/mL). Subsequently, 500 µL of 10% SDS in 0.01 M HCl was added to dissolve the formed formazan crystals and the plates were kept overnight at 37 °C, 5% CO_2_ and in the dark. The next day the absorbance of each well was measured at 595 nm. From this, 100% cell viability was considered that of the negative control (cells without treatment).

### 4.8. Statistical Analysis

Data are presented as mean ± standard error of the mean (SEM) of *n* animals/wells performed. Normal distribution of the data was evaluated using the Shapiro–Wilk (*n* < 50) or Kolmogorov–Smirnov (*n* ≥ 50) test. Comparisons between groups were assessed by an ANOVA–Scheffé or a Kruskal–Wallis test. A value of *p* < 0.05 was considered significant. Statistical analysis was performed using IBM SPSS Statistics 20.0 software (International Business Machines, Armonk, NY, USA). Microsoft Office Excel and PowerPoint 2016 (Microsoft, Redmond, WA, USA) were used to create the artwork and illustrations.

## 5. Conclusions

P-quercetin protects against cisplatin damage via intraperitoneal, but not by the oral one, even though the formulation is absorbed by both routes. The nephroprotection of quercetin against cisplatin could be related to the concentration of some of its metabolites as they pass through the kidney. In our cytoprotection experiments, the compounds quercetin, quercetin 3-*O*-glucoside, rutin and isorhamnetin did not appear to protect against cisplatin cytotoxicity in tubular cells. More experiments are needed for tamarixetin to check the cytoprotection profile observed. In contrast, quercetin 3-*O*-glucuronide exerts a moderate cytoprotective effect, preventing the cytotoxicity and antiproliferative effect of cisplatin in renal tubular cells, therefore being a possible candidate for future nephroprotection strategies in vivo.

## Figures and Tables

**Table 1:** Higher non-toxic concentrations of quercetin metabolites in HK-2 cells. The data were obtained by cell viability tests (MTT assay; Figure S1, Supplementary Material).

	Maximum Non-Toxic Concentration (μM) in HK-2 Cells
Isorhamnetin	1.56
Quercetin	12.5
Quercetin 3-*O*-glucoside	0.78
Quercetin 3-*O*-glucuronide	25
Rutin	25
Tamarixetin	12.5

**Table 2:** Higher non-toxic concentrations of quercetin metabolites in NRK-52E cells. Data were obtained by cell viability assays (MTT assay; Figure S3, Supplementary Material).

	Maximum Non-Toxic Concentration in NRK-52E Cells (μM)
Quercetin	25
Quercetin 3-*O*-glucuronide	200
Tamarixetin	12.5

## Data Availability

The data presented in this study are available on request from the corresponding author.

## Supplementary Materials

The following are available online, Figure S1: Quercetin metabolites safety assays in HK-2 cells for 24 h of treatment, Figure S2: Isorhamnetin, quercetin 3-*O*-glucoside and rutin efficacy assays in HK-2 cells for 24 h against the cytotoxicity of cisplatin, Figure S3: Quercetin, quercetin 3-O-glucuronide and tamarixetin safety assays in NRK-52E cells for 24 h of treatment. Table S1: Mass spectral data and tentative identification of the compounds detected in the analyzed urine samples.

## Sample Availability

Not available.
