# Supplementary material for: Protective Effect of Quercetin 3-O-Glucuronide against Cisplatin Cytotoxicity in Renal Tubular Cells
Source: Molecules. 2022 Feb 15;27(4):1319. doi: 10.3390/molecules27041319 (PMC8880316; doi:10.3390/molecules27041319)
Supplement: Supplementary file 1 [file molecules-27-01319-s001.zip › molecules-1583638-supplementary.pdf]

molecules

MDPI

Supplementary Material

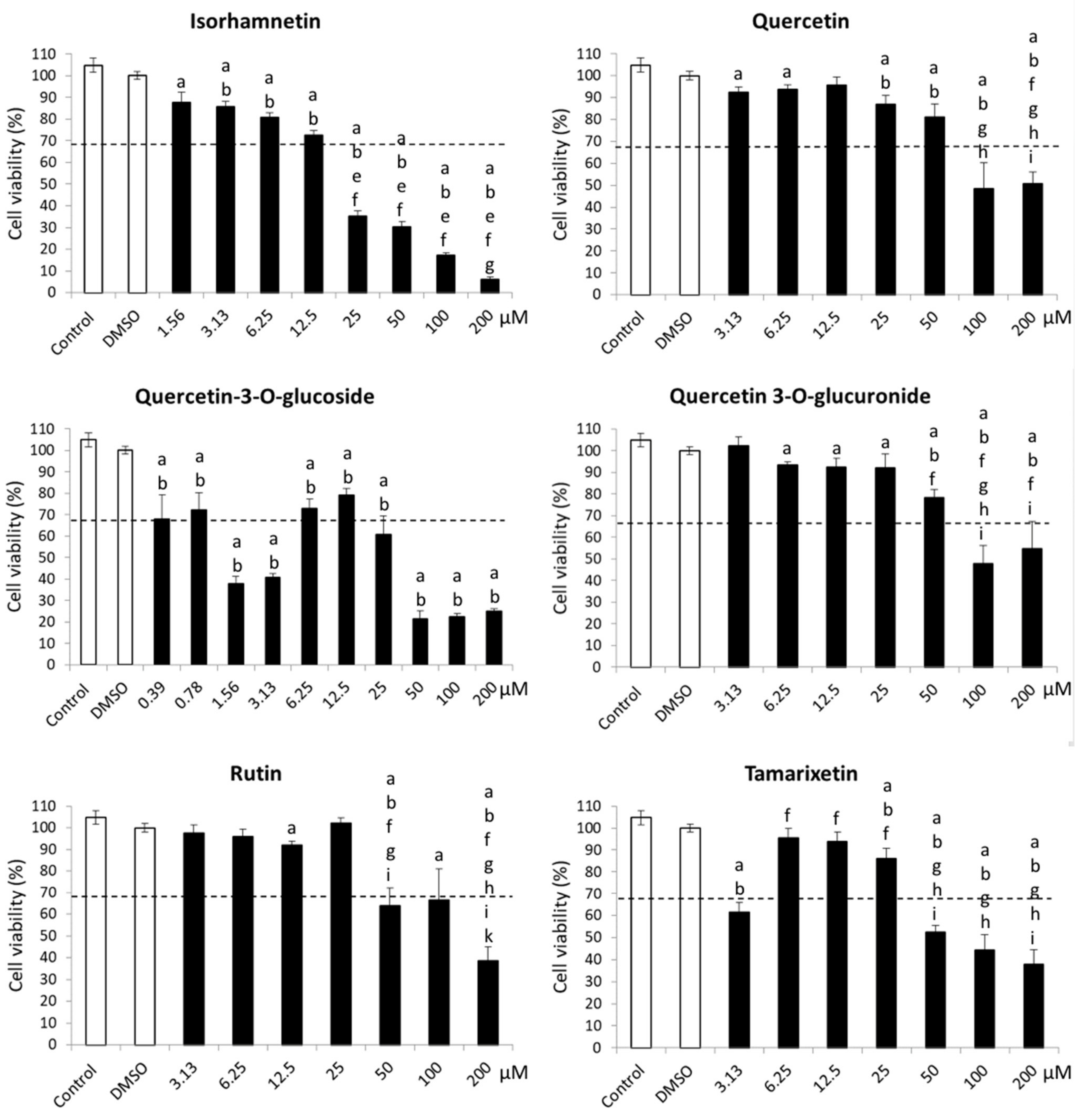

Figure S1. Quercetin metabolites safety assays in HK-2 cells for 24 hours of treatment. The MTT technique was used to estimate cell viability. Values are expressed as the mean ± SEM. Significant differences ($p < 0.05$): a vs. Control; b vs. DMSO; c vs. 0.39 υM; d vs. 0.78 µM; e vs. 1.56 µM; f vs. 3.13 µM; g vs. 6.25 µM; h vs. 12.5 µM; i vs. 25 µM; j vs. 50 µM; k vs. 100 µM. The dotted line represents the state of the cells before treatment (time 0h). DMSO: dimethyl sulphoxide.

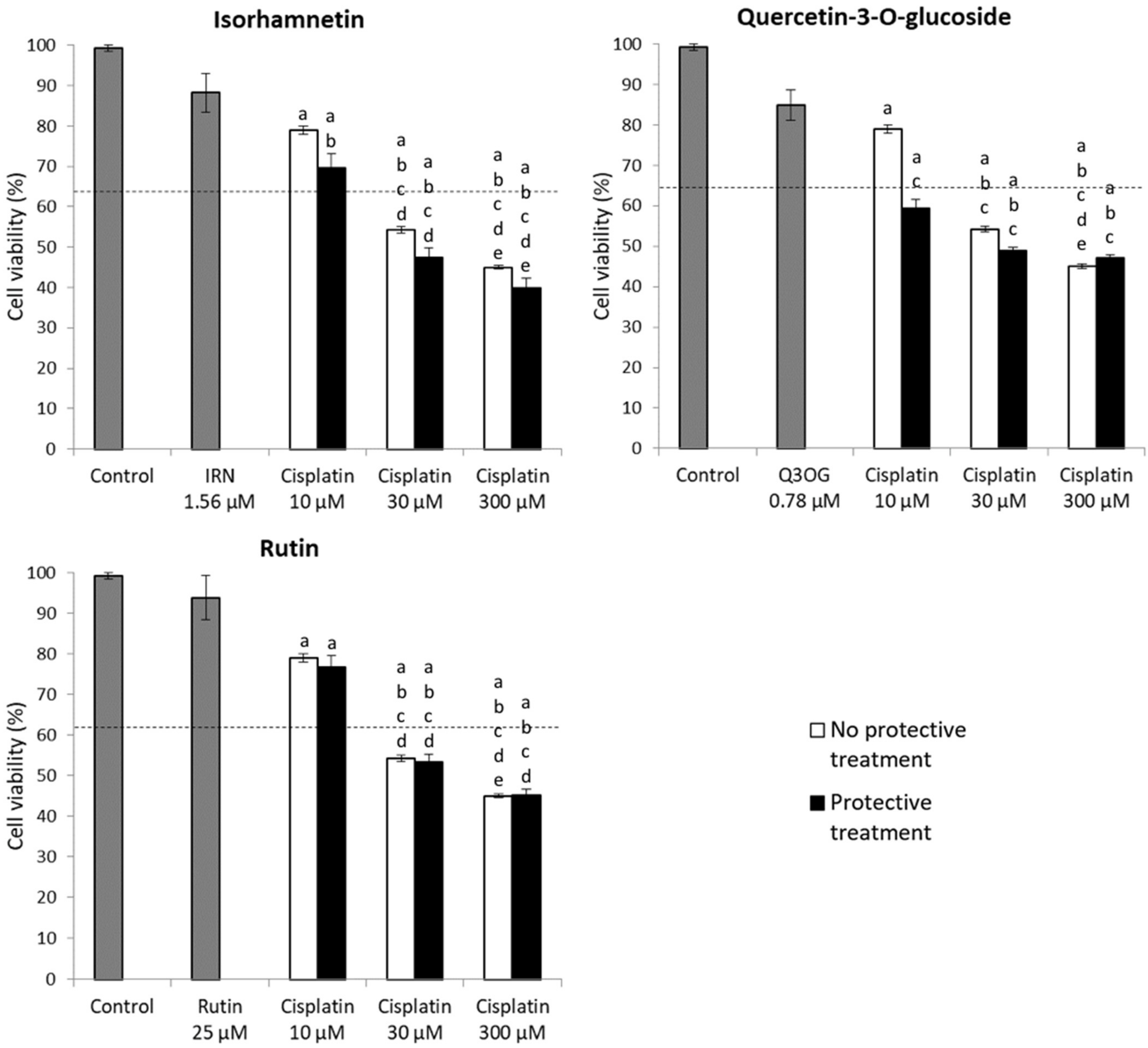

Figure S2. Isorhamnetin, quercetin 3-O-glucoside and rutin efficacy assays in HK-2 cells for 24 hours against the cytotoxicity of cisplatin. The cells were pretreated for 6 hours with the corresponding metabolite and subsequently co-treated for 18 hours with the metabolite and 10, 30 and 300 µM cisplatin, respectively. The MTT technique was used to estimate cell viability. Values are expressed as the mean ± SEM. Significant differences ($p < 0.05$): a vs. Control; b vs. Protective treatment alone; c vs. Cisplatin 10 µM; d vs. Cisplatin 10uM + Protective treatment; e vs. Cisplatin 30 µM. The dotted line represents the state of the cells before treatment (time 0h). IRN: isorhamnetin; Q3OGls: quercetin-3-O-glucoside.

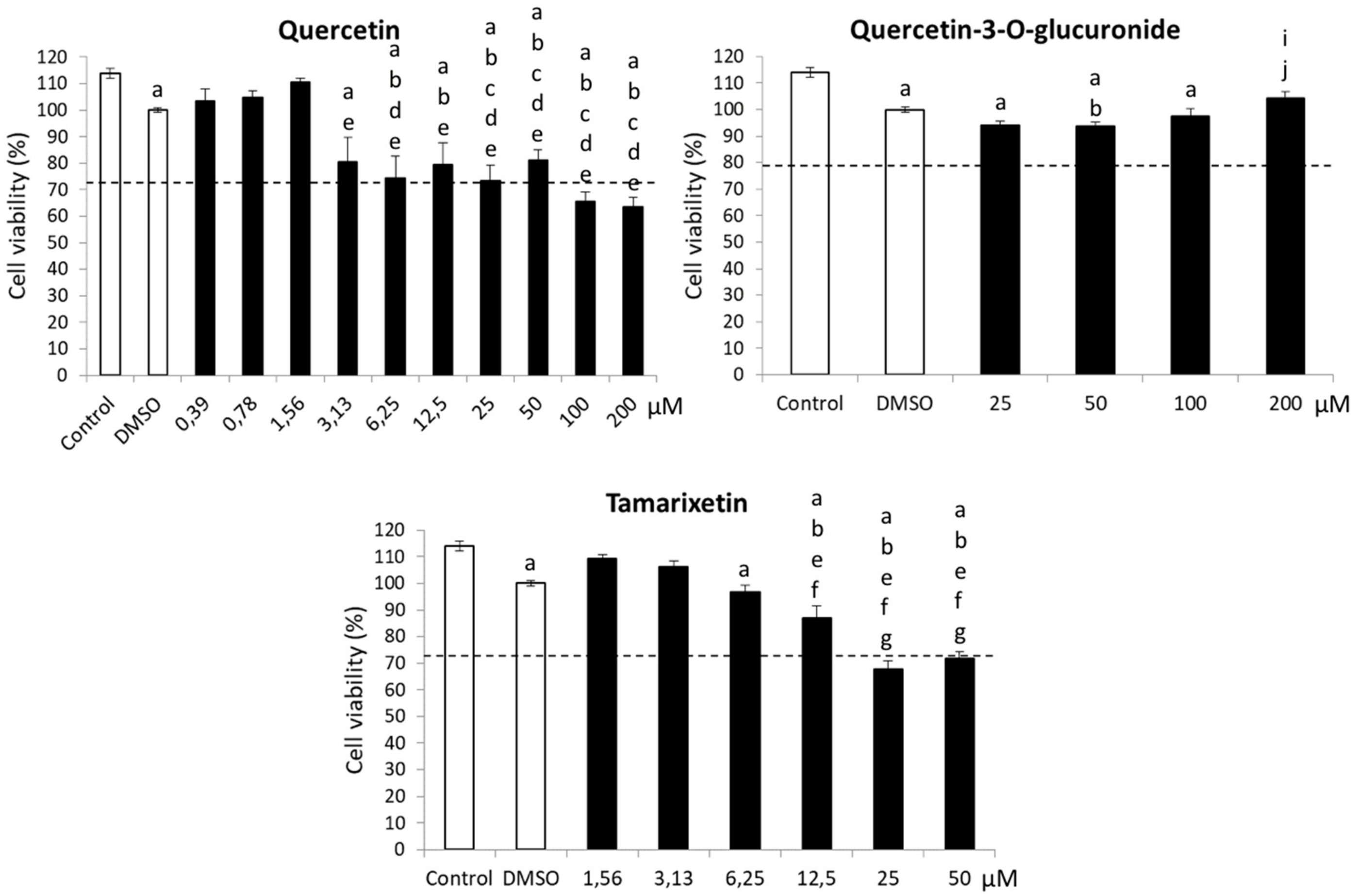

Figure S3. Quercetin, quercetin 3-O-glucuronide and tamarixetin safety assays in NRK-52E cells for 24 hours of treatment. The MTT technique was used to estimate cell viability. Values are expressed as the mean ± SEM. Significant differences ($p < 0.05$): a vs. Control; b vs. DMSO; c vs. 0.39 υM; d vs. 0.78 µM; e vs. 1.56 µM; f vs. 3.13 µM; g vs. 6.25 µM; h vs. 12.5 µM; i vs. 25 µM; j vs. 50 µM; k vs. 100 µM. The dotted line represents the state of the cells before treatment (time 0h). DMSO: dimethyl sulphoxide.

Table S1. Mass spectral data and tentative identification of the compounds detected in the analyzed urine samples.

**Peak**	**Pseudomolecular ion [M-H]⁻ (*m*/*z*)**	**MS/MS (*m*/*z*)**	**Tentative identity**
1	243	201, 183,153	Protocatechuic acid derivative
2	231	187, 151	Hydroxyphenylacetic acid sulfate
3	557	477, 301	Quercetin glucuronide sulfate
4	153	135,109	Protocatechuic acid
5	667	491	Methylquercetin diglucuronide
6	381	-	Quercetin sulfate
7	653<br>463	477<br>301	Quercetin diglucuronide<br>Quercetin glucose
8	557	477, 301	Quercetin glucuronide sulfate
9	477	301	Quercetin glucuronide
10	571	491, 315,301	Methylquercetin glucuronide sulfate
11	491<br>557	301<br>477, 301	Methylquercetin glucuronide<br>Quercetin glucuronide sulfate
12	491	315, 301	Methylquercetin glucuronide
13	381	301	Quercetin sulfate
14	381	301	Quercetin sulfate
15	491	315, 301	Methylquercetin glucuronide
16	381	301	Quercetin sulfate
17	395	315, 301	Methylquercetin sulfate
18	395	315, 301	Methylquercetin sulfate
19	315	301	Methylquercetin
